# Significance of Blood and Cerebrospinal Fluid Biomarkers for Alzheimer’s Disease: Sensitivity, Specificity and Potential for Clinical Use

**DOI:** 10.3390/jpm10030116

**Published:** 2020-09-08

**Authors:** Cristina d’Abramo, Luciano D’Adamio, Luca Giliberto

**Affiliations:** 1 The Litwin-Zucker Center for Alzheimer’s Disease & Memory Disorders, Institute of Molecular Medicine, Feinstein Institutes for Medical Research, Northwell Health System, Manhasset, NY 11030, USA; cdabramo@northwell.edu; 2 Rutgers Alzheimer’s Disease & Dementia Research Center (RUADRC), Rutgers Brain Health Institute, Rutgers University, Piscataway, NJ 08854, USA; luciano.dadamio@rutgers.edu; 3 Northwell Health Neuroscience Institute, Northwell Health System, Manhasset, NY 11030, USA; 4 Donald and Barbara Zucker School of Medicine, Hofstra University, Hempstead, NY 11549, USA

**Keywords:** Alzheimer’s disease, biomarkers, cerebrospinal fluid, blood, amyloid, tau, soluble TREM2, NfL, Multiplex, SiMoA

## Abstract

Alzheimer’s disease (AD) is the most common type of dementia, affecting more than 5 million Americans, with steadily increasing mortality and incredible socio-economic burden. Not only have therapeutic efforts so far failed to reach significant efficacy, but the real pathogenesis of the disease is still obscure. The current theories are based on pathological findings of amyloid plaques and tau neurofibrillary tangles that accumulate in the brain parenchyma of affected patients. These findings have defined, together with the extensive neurodegeneration, the diagnostic criteria of the disease. The ability to detect changes in the levels of amyloid and tau in cerebrospinal fluid (CSF) first, and more recently in blood, has allowed us to use these biomarkers for the specific in-vivo diagnosis of AD in humans. Furthermore, other pathological elements of AD, such as the loss of neurons, inflammation and metabolic derangement, have translated to the definition of other CSF and blood biomarkers, which are not specific of the disease but, when combined with amyloid and tau, correlate with the progression from mild cognitive impairment to AD dementia, or identify patients who will develop AD pathology. In this review, we discuss the role of current and hypothetical biomarkers of Alzheimer’s disease, their specificity, and the caveats of current high-sensitivity platforms for their peripheral detection.

## 1. Introduction

### 1.1. Clinicopathological Definition of Alzheimer’s Disease

Alzheimer’s disease (AD) is a neurodegenerative disorder, characterized by progressive cognitive impairment, affecting the domains of memory, speech, praxis, awareness and executive function, representing a decline from the prior functional state, with a relentless course [1]. It is the most common cause of dementia. Along with the destructive nature of the disease, which affects patients and their families, AD represents an enormous burden on public health. An estimated of 5+ million Americans have AD, and the prevalence worldwide reaches almost 60 million; the diseases hits the elderly population the most, with 11% of individuals 65 and older affected by the disease [2,3]. Two thirds of AD patients are women, implying hormonal, lifestyle and genetic factors as co-causative elements [2,4,5,6,7,8,9,10,11,12,13,14,15]. AD is the sixth leading cause of death in the US [16] and mortality due to AD has steadily increased in the last 20 years, in contrast to opposite trends for other chronic diseases, such as cardiovascular, cancer, HIV and more. The socio-economic burden of the disease, on patients, families and caregivers at large, and on society, is unparalleled; AD is the disease that contributes most to disability and poor health in the US, reducing life expectancy and productivity of the affected individuals and the wellbeing of caregivers, who often have to give up jobs and careers to care for their loved ones [17,18,19]; this is often disproportionally affecting women too [20,21,22]. Finally, AD has a tremendous economic impact: the sole health care costs, variably covered by insurances (including Medicare and Medicaid) or out of pocket, to care for this disease, is in excess of 230 billion [23]. Age-matched comparisons show that individuals affected by dementia had 3–4x the annual health care expenses than those not affected. It is estimated that these costs will reach, in 2050, 1 trillion dollars (2020 Alzheimer’s Disease Facts and Figures. Available online: https://www.alz.org/media/Documents/alzheimers-facts-and-figures.pdf. Accessed on 29 July 2020), a figure comparable to the US budget deficit in 2020. Interestingly, these numbers should probably be corrected upward, as the disease is often under-recognized and under-reported. This is particularly relevant to our discussion, given current limitations in performing early and accurate diagnosis.

The pathological diagnosis of AD relies on the assessment of specific brain features [24,25,26,27], based on the presence, morphology and density of lesions and their topographic distribution, in the appropriate clinical setting. Alzheimer’s brains are characterized by a spatiotemporally defined accumulation of amyloid beta (Aβ) plaques, neurofibrillary tangles (NFT), of hyperphosphorylated and aggregated tau and neuronal loss [25,28,29]. Aβ accumulates mostly as the Aβ peptide ending at residue 42, which is considered the most toxic and most prone to aggregation [30]; tau accumulates as a hyperphosphorylated and conformationally altered form, in both its 3- and 4-repeats variants [31]. The trajectory of protein accumulation is relevant. It is speculated that Aβ accumulates first; this is followed by the hyperphosphorylation, conformational changes and aggregation of tau; finally, neuronal loss ensues. 

The link between these three features is, to some degree, still loose. Both Aβ [30,32] and tau [33,34] are per se neurotoxic; Aβ accumulation can trigger tau accumulation [35] but, in several models, tau can independently aggregate and cause neurodegeneration [36]. Furthermore, amyloid deposition, by pathological and imaging staging (amyloid positron emission tomography—PET), does not topographically and functionally correlate with the clinical cognitive decline seen in our patients, while tau does, and there are cases of aged individuals who have amyloid deposition in their brain, but no tau and no clinical dementia [25,37,38].

A significant degree of co-pathology, not strictly specific of AD, is common in these brains [39]. This includes the presence of activated microglia and reactive astrocytes, which appear initially around neuritic plaques, and increase in density and activation in proportion to the neuronal damage and the presence of tau pathology [40,41,42]; vascular alterations, in the form of amyloid deposition in the blood vessels (CAA: cerebral amyloid angiopathy); accumulation of other structural proteins in neurons, such as actin and actin-associated proteins in the form of Hirano bodies [43]; granulovacuolar degeneration (GVD), a feature often associated with the initial hyperphosphorylation and aggregation of tau in the hippocampus [44]. Argyrophilic grains of 4-repeat tau can also be found in neuronal dendrites, near oligodendrocytes inclusions and seems to be relevant to disease progression [45].

In some AD cases, specific neuronal populations are vulnerable to develop Lewy body pathology of α-synuclein [43,46], which is a signature of Parkinson’s disease (PD), mostly at the axonal level, in cortico-limbic regions, olfactory bulbs and substantia nigra. Finally, TAR DNA-binding protein 43 (TDP-43) pathology, more commonly found in cases of frontotemporal lobe degeneration (FTD), can be present in AD, in the amygdala, hippocampus and eventually in the neocortex and more diffusely [47,48], being not specific of AD, but underlying the vast array of cellular dysfunction present in this disease, which can affect RNA-related metabolism as well [49]. The common path, which is likely consequent to all the above changes, is the dramatic loss of synapses, which precedes neuronal loss, and mostly follows the distribution of tau pathology [43,50,51,52].

The routine clinical diagnosis of possible or probable AD is currently based on the identification of symptoms by the clinician, mostly Neurologists, Geriatricians, Psychiatrists and Primary Care Physicians, followed by more in-depth assessment by Neuropsychology testing and using the tools available in clinical practice, e.g., Magnetic Resonance Imaging (MRI) of the brain and common blood works. Once AD is suspected, these tools allow to rule out secondary dementia and other, different pattern of primary dementia. On a more sophisticated level, the analysis of the cerebrospinal fluid (CSF), the use of PET imaging and genetics (in familial cases) may allow us to obtain a definitive diagnosis of AD in vivo: due to cost and perception of invasiveness, this is often limited to Academic centers or in the context of clinical trials.

Ideally, we should diagnose AD, and every dementia, as early as possible. Mild cognitive impairment (MCI) is a prodromal phase of dementia, where the patient shows symptoms of cognitive changes but maintains good functionality. This is a moment where the application of biomarkers is paramount and significant therapeutic intervention may still possible. Unfortunately, there is a significant underappreciation of the disease, which is missed in more than 20% of the cases. On the other hand, lack of familiarity with alternative diagnoses, leads to overdiagnosis of AD in more than 25% of the cases, where potentially curable conditions are instead the underlying cause of the cognitive and functional decline [53,54,55,56].

### 1.2. Pathogenesis

The majority of our understanding of the pathogenesis of AD derives from the identification of key genes involved in the etiology and pathology of the disease. In a small percentage of the cases, the disease is inherited as autosomal dominant (FAD: familial AD), with an early age of onset, as early as the ‘40s, and a faster progression [57]. FAD is caused by mutations of one of three genes, Amyloid Precursor Protein (APP), Presenilin 1 (PS1) or Presenilin 2 (PS2). APP is the precursor of the amyloid peptide, Aβ, which is generated from APP by two sequential cleavages: first, the beta-secretase activity of BACE1 (beta-site amyloid precursor protein cleaving enzyme 1) generates a peptide of ~99 amino acids (C99); subsequently, the activity of the gamma-secretase, a multi-peptide protease, progressively cleaves C99 to generate the Aβ peptide ending at residues 40 or 42. The gamma secretase complex is composed of PS1 or PS2 and three other proteins, nicastrin, Aph1 and Pen2 [58]. Mutations in APP mostly lead to increase affinity of APP itself for BACE1, with increased generation of C99. Mutations of PS1 and PS2, which result in an actual loss of function mutations of gamma-secretase [59,60,61,62], lead to increased generation of the Aβ peptide ending in residue 42. This is recognized as the most toxic of the Aβ peptides [63]. About 95% of AD cases are sporadic (SAD) and have a later onset (after 65 years of age): although SAD cases lack a Mendelian cause, variants of genes such as Apolipoprotein E (*APOE*) [64,65], and triggering receptors expressed on myeloid cells 2 (TREM2) [66] are known to increase the risk of developing SAD. It is not clear what the initial trigger to Aβ and tau accumulation is in the sporadic disease. At the subcellular and molecular level, the complexity of Aβ and tau accumulation and their specific contribution to pathology has been extensively studied, and is beyond the scope of the current publication. The general understanding is that both amyloid and tau oligomers have the ability to disrupt several of the essential functions of neurons, and possibly other brain cells, i.e., metabolic and mitochondrial activity [67,68], synaptic communication [69,70], membrane integrity [71], membrane pore formation with ions leakage [72,73], protein trafficking and degradation [74,75]. Both Aβ and tau possess the ability to trigger a cascade of functional changes in neurons, microglia, astrocytes and oligodendrocytes, which results in neuronal dysfunction, synaptic loss, innate immune system activation, inflammation, blood-brain barrier impairment and neurodegeneration [39,76]. Relevant to our discussion, the immune system is activated (early) in AD, and may contribute, by means of ineffective clearance of Aβ and tau by the microglia, to the diffusion of such pathology to neighboring or connected areas [77,78], and inflammation per se is a trigger to further production of Aβ and activation of kinases to hyper-phosphorylate tau [79], cascading more neuropathology.

### 1.3. The (Amyloid, Tau, Neurodegeneration) ATN System

Stemming from the above definitions, pathological correlates and pathogenic theories, AD can be viewed as the combination of specific brain pathology, Aβ and tau, and neurodegeneration, which eventually results in the clinical presentation. Recent advances in brain imaging and more sensitive and specific means to detect Aβ and tau in biological fluids, associated with our ability to detect loss of brain matter, metabolism or function, have allowed for development of a classification and staging system for AD, the ATN system [80], which is routinely revised [81], to include the everchanging horizon of new biomarkers available. Patients are classified by positivity to markers of amyloid deposition (A), tau load (T) and neurodegeneration (N). Amyloid markers include the positivity to Amyloid PET (several tracers are now available) or reduced levels of cerebrospinal fluid (CSF) Aβ42, Aβ40 or of the Aβ42/40 ratio (see below). T is defined by the high levels of total or phosphorylated tau in the CSF (see below), although the new available tau PET tracers may soon be included in the criteria. Finally, N is related to the neurodegenerative aspect, and can be defined by loss of volume/activity in specific areas of the brain by magnetic resonance imaging (MRI, e.g., NeuroQuant, [82,83]), fluorodeoxyglucose (FDG)-PET or by the presence of CSF biomarkers of synaptic or neuronal loss, e.g., Neurofilament Light chain (NfL).

This system has been a useful tool for many biomarkers studies, both in biological fluids and imaging, remaining the “benchmark” for AD diagnosis and prognosis determination. Importantly, the ATN system can be extremely useful to identify and correctly classify individuals who may have amyloid deposition but no neurodegeneration (A+T-N-) and vice versa (A-T-N+); this is extremely important when studying pathophysiological correlates and in longitudinal assessments. As hinted above, a good proportion of the ageing population will show deposition of amyloid in their brain, but no clinical dementia; we know that this amyloid may not be the same amyloid that accumulates in AD proper [84], hence not cause the disease, but also other factors may not be present in these individuals that predispose to tau accumulation and neurodegeneration. On the other hand, neurodegeneration and dementia may occur because of pathology other than Alzheimer’s, e.g., vascular, TDP43, alpha-synuclein, etc. If rigorously determined and applied to our studies, the ATN system will be invaluable, as it prescinds from the subjective interpretation of the clinical status of the patients, and is purely descriptive of their biomarkers status.

It must be noted, though, that not all the pathophysiological events and biomarkers in AD can be inscribed in the ATN definition, hence we will be presenting a wider paradigm, that includes, for example, inflammation.

### 1.4. Treatment Options

No disease-modifying treatment is available as of today, despite decades of efforts aimed to curb the pathological changes of the disease [85,86,87]. The widest attempts to tackle the disease have been directed against the production/accumulation of Aβ and have overall been underwhelming [85]. Reducing its production via inhibition of BACE or gamma-secretase has not been effective, resulting in potential side effects, included hastening the cognitive decline [86]; active vaccination efforts against Aβ has also not been successful, mostly due to cases of dramatic adverse events, in the form of hemorrhagic encephalitis [88], despite some cognitive benefits in the survivors [89]. Alternative active vaccinations strategies, aimed at inducing a wide reactivity to Aβ without the excessive inflammatory response, have been tested [90,91] with scarce success. Passive immunization against Aβ was also tainted by questionable clinical efficacy and concerns of serious side effects [92,93,94,95], but efforts are well under way: at the moment that we write this review, a monoclonal antibody against amyloid has been submitted for U.S. Food and Drug Administration (FDA) expedited approval as a modifying drug against AD, with some skepticism among the scientific community in respect to the available efficacy data [95] (NCT02477800). Tau has been the other logical therapeutic target. Current strategies are directed to target total tau, which is thought to accumulate intra- and extracellularly, and may spread from cell to cell, aggregating and getting hyperphosphorylated, contributing to neurodegeneration. Antibodies targeting extracellular tau have been in clinical trial now for some time, and we are all bracing for the upcoming results. Of note, this strategy has so far failed in progressive supranuclear palsy (PSP) cases with tau pathology (NCT03068468). An active immunization approach against tau is currently under way as well [96,97].

What clinicians are left with is the use of symptomatic treatments. Acetylcholine deficits are well recognized in AD [98], and are the basis for acetylcholine esterase inhibitors, which can ameliorate quality of life. Memantine, a noncompetitive N-methyl-D-aspartate-receptor antagonist, has also shown some benefit in maintaining cognitive function, alone and in association with acetylcholine esterase inhibitors. In all, unfortunately, the enthusiasm for these agents is very low, due to low efficacy and possible adverse events, such as behavioral changes, heart arrhythmias, loss of appetite and weight, which in the elderly population are a major concern.

The neuropathological changes described above are projected to begin decades before the presentation of symptoms. This makes any medical intervention, even in the prodromal stages, a “too little-too late” approach, with few chances of being meaningful. The challenges in AD are, in our opinion, 2-fold; first, defining the actual pathogenesis of the disease, by far not yet clear; second, be able to identify the risk to develop the disease early enough so that disease-modifying intervention can be effective.

### 1.5. Definition of AD Biomarkers and Diagnostic Challenges

What is a biomarker? What is an adequate and useful diagnostic biomarker for Alzheimer’s disease? While it is fairly easy to respond to the first question, it is very difficult to answer the second. The reason for the latter is that, save for the genetically determined cases of AD (a minority of the cases), in the absence of a univocal and comprehensive pathogenic pathway, it is unlikely to be able to diagnose AD with certainty in the living adult in a time frame that would be useful for significant intervention.

A biomarker is a “defined characteristic that is measured as an indicator of normal biological processes, pathogenic processes, or responses to an exposure or intervention, including therapeutic interventions” (FDA-NIH Biomarker Working Group. BEST (Biomarkers, EndpointS, and other Tools). Available online: https://www.ncbi.nlm.nih.gov/books/NBK326791/. Accessed on 29 July 2020). This definition includes both a diagnostic and prognostic element. The current literature about AD biomarkers is extensive and rich of analyses and reviews. The popular website, Alzforum, has its own dedicated meta-analysis page (Alzforum. Available online: https://www.alzforum.org/alzbiomarker. Accessed on 29 July 2020).

AD biomarkers are quite reliable indicators of disease presence, topographical extension and stage. Levels of Aβ, total tau (t-tau), and phosphorylated tau (p-tau) are concurrent with AD pathology [99,100] and indicate its presence; at this stage amyloid [101] and tau PET imaging [102] define the topographical extension of such pathology, while MRI [103] and FDG-PET [104] define the topographical severity of the neurodegenerative process; finally, markers such as p-tau, NfL [105] and YKL40 [106] can be useful, and early, peripheral indicators of disease stage.

We can use a combination of fluid and imaging biomarkers to complement the clinical investigation in our patients, and fulfill the current diagnostic criteria [107,108,109,110] with ease. One caveat is that while these criteria can work well in research settings [111,112], the reality of clinical practice is tainted by the reluctance of patients to have invasive testing done (CSF) and by the lack or insurance support for the more “fancy” imaging [113]. Fortunately, as described below, the rapid evolution of detection techniques is allowing to reach remarkable results from blood biomarkers, which would improve access for patients.

Another element must be taken in consideration when defining a useful biomarker: time. The concept of early diagnosis has become crucial in neurodegeneration, since protein aggregation and accumulation might only reflect the last stage of the pathogenic cascade, a point of no return in the clinical manifestation and progression of the diseases, where therapeutic interventions have so far been ineffective. As mentioned above, the exact succession of pathogenic events in AD is still unclear; Aβ and tau may be causative, but subtler prior changes in neuronal connectivity, metabolic patterns and inflammation could represent an early disruption of neuronal function, leading to secondary accumulation of Aβ and tau [36]. In this context, an early diagnosis should go beyond Aβ and tau. A “late” biomarker’ profile may also be tainted by ongoing and long-standing pathology, and by other factors related to ageing, e.g., brain trauma, infections, microvascular disease/strokes, and co-morbidity with other frequent neurodegenerative conditions such as PD [114]. The clinical presentation of FAD and SAD is similar, except for the age of onset. For FAD mutations carriers, preclinical diagnosis is possible by genetic analysis; the more common SAD cases, instead, remain elusive until clinically evident. Hence, the challenge of procuring an early preclinical diagnosis of disease or susceptibility to it.

With these limitations in mind, the science of AD biomarkers has evolved tremendously in the last decade, and we are looking now at the possibility to have reliable peripheral non-invasive, scalable and affordable biomarkers of AD pathology and disease stage.

We will review below the current, most promising fluid (blood and CSF) biomarkers available, discuss the pathophysiologic correlate of each, their clinical significance, their general applicability in the clinical and research setting. We will then take tau as an example of biomarker detection complexity, with blessings and caveats, stemming from the need to accurately validate data emerging from new ultra-sensitive platforms, in light of their biochemical limitations.

Importantly, we have divided the below presentation in biomarkers that are specific of AD, where their appropriate combination in biological fluids defines the disease, from biomarkers that are representative of neurodegeneration or associated pathology, such as synaptic and neuronal loss, inflammation, vascular disease and others. The latter biomarkers may be present in neurodegenerative diseases different from AD, in head trauma, in stroke and infections, hence they are not specific for AD. They are, though, extremely useful to follow, and at time predict, AD progression and presentation. Their precise causative role in the neurodegenerative process is still unknown, hence we will present the current known hypotheses in descriptive fashion.

## 2. Biomarkers of AD Pathology

### 2.1. Aβ

Given its pathophysiological role, Aβ has revealed itself as the major biomarker of the disease, in addition to being one of main therapeutic targets so far. Over the last 30+ years, exponential efforts have demonstrated that CSF (and more recently blood) levels of Aβ (1) are reduced in AD compared to non-demented controls [115], (2) are decreased in mild cognitive impairment (MCI) patients compared to normal controls [116], (3) the trend of Aβ decrease is predictive of the clinical progression of MCI to AD and preclinical AD [117,118] to MCI. Similarly, and (4) the Aβ42/40 ratio is reduced in MCI and AD, with better clinical correlations to than total Aβ [119,120].

What is the pathogenic significance of Aβ reduction in biological fluids? Why is the Aβ42/40 ratio going down as the disease progresses? Is there ever a time, in the preclinical phases of the disease, when Aβ is instead increased in biological fluids, possibly marking a pivotal moment where a therapeutic intervention would still be effective?

In FAD, APP mutations lead to an increased overall production of Aβ, since the amyloidogenic cleavage of APP is favored. Mutations in PS1 or PS2 result instead in a preferential cleavage of C99 to form the Aβ42, while in reality the overall amount of Aβ may be reduced, since these are loss of function mutations [121]. In either case, there is an absolute or relative abundance of Aβ42 released in the brain parenchyma, which has a high tendency to aggregate and deposit as oligomers, fibrils and plaques in a process of nucleation [122]. Furthermore, BACE cleavage is seldom limited to residue #1 of Aβ; the amyloid peptide often starts at residue #3 or #11, a glutamate residue (E), which tends to be cyclized [123,124] as pyro-glutamate (pE). The resulting AβN3(pE) and AβN11(pE) peptides also have high tendency to aggregation, adding to the stickiness of Aβ42, and have been considered one of the first seeding species of Aβ [125]. In this context, it is easy to understand how the parenchymal sequestration of Aβ results in lower level of the soluble molecule available to diffuse to the interstitial fluid, CSF and blood, hence the low levels thereof. Further decrease of Aβ in the periphery may also be related to the progressive and massive loss of neuronal cells along the progression of the disease, as neurons are the highest manufacturers of the peptide [126]. Finally, other factors such as inflammation, infection or perturbation of Aβ clearance mechanism may affect its peripheral levels, though unlikely to have a chronic effect [127].

Other conditions show the opposite; in traumatic brain injury (TBI), for example, CSF and plasma levels of Aβ1-42 increase steadily after the acute event, and normalize with recovery in weeks [127,128,129]; in some cases, an increase of APP itself was noted when sampling intraventricular CSF [130]. These data tend to correlate with other markers of neuronal damage, i.e., the release of total tau, neuronal specific enolase (NSE) and APOE, but the actual mechanism of shedding is unclear. Notably, axonal injury after TBI is associated with redistribution of APP towards the axons and increased processing to generate Aβ, explaining part of the CSF levels [131,132].

Interestingly, whereas Aβ accumulation in the brain results in a reduction in the periphery, tau accumulation in the brain parallels its peripheral abundance [115] (discussed below). Tau accumulation follows, in time, Aβ accumulation. Can this time difference (maybe decades) explain the dichotomy? If so, is there a moment in time in AD pathogenesis when Aβ levels are actually increased or normal in the periphery? The current data on autosomal dominant AD show initial amyloid-PET abnormalities ca. 22 years prior the expected symptom onset (EYO-22) and CSF Aβ reduction (and tau increase) a few years later (EYO-19 to -14) [133]. This series of events signifies that (1) sensible Aβ deposition in the brain happens quite early in the pathogenesis, (2) current CSF (and blood) biomarkers follow brain changes by several years, and (3) measures of neuronal damage (peripheral tau, etc.) do not fall far behind peripheral Aβ changes. This leaves open the question of what happens even earlier to peripheral Aβ, is it ever elevated? Is that the ideal moment where anti-Aβ therapeutic strategies would be most effective? Only longitudinal studies with a very early CSF and blood sampling will be able to answer these questions.

A recent study in a Down syndrome (DS) cohort [134], with CSF sampling from age 30 to 61, showed Aβ42 reductions which was proportional to age and cognitive decline (from cognitive stable, to MCI to AD), with higher levels than those reported in corresponding older non-DS controls, MCI and AD [135], but did not have a non-DS age-matched control group. In another study [136], CSF levels of Aβ peptide were found to be elevated from 8 to 54 months of age in DS children, while tau was low, likely due to Aβ overproduction in the absence of significant parenchymal sequestration and neuronal loss at that age.

CSF. Sampling CSF by lumbar puncture (LP) is the most direct way to obtain biomarkers that reflect what happens in the brain. Being invasive, most patients do not feel comfortable undergoing a spinal tap, although the rate of complications, i.e., post-LP headache, bleeds or infections is very low [137]. CSF levels and variations of Aβ have long been the hallmark features of AD-type dementia. The literature on the matter is extremely extensive and will not be reviewed here. We will limit our discussion to the clinicopathological relevance of Aβ in CSF. The first attempts at measuring soluble APP-derived fragment in the CSF of AD patients were carried out by D. Selkoe and S. Younkin [138] and B. Frangione [139] labs, determining the reduction of soluble APP (sAPP) fragments in the CSF of AD patients. Soon after, scientists were able to map the Aβ region and retrieve the peptide in the CSF [140]. This led to the first studies looking at differences between AD and control subjects in soluble APP and Aβ peptides [141,142,143,144,145], with notable differences likely due to detection methods and populations [146,147].

CSF levels of Aβ 42 are generally lower in AD compared to control subjects, and have been seen to decline with disease progression [135,147,148,149,150]. Aβ1-42 levels can be reduced as much as 50% in patients with AD compared to control subjects [151]. There seems to be, though, significant overlap of total Aβ1-42 levels between AD and non-AD dementia, such as vascular dementia, Parkinson’s disease dementia (PDD), Lewy body disease (LBD), frontotemporal lobe degeneration (FTD) and even amyotrophic lateral sclerosis (ALS), and this may be due to population selection, stage of disease or overlap of pathogenic events in advanced disease [152,153,154,155,156,157,158].

In the pre-dementia stages, both amnestic MCI (aMCI), the most frequent precursor of AD, and non-amnestic MCI, showed low levels of Aβ1-42 [154,159,160,161,162] compared to control subjects, also with significant overlap.

In all, the use of Aβ1-42 alone does not seem to be too promising for differentiating AD from non-AD dementia and less so at the MCI stage, due to heterogeneity and overlap. As mentioned above, the pathophysiology of APP processing in AD is characterized by an imbalance of the 1-42 over the 1-40 species of Aβ, with preferential deposition of the former in the brain. Additionally, the gamma-secretase complex cleaves APP in a sequential fashion, generating progressively shorter fragments, like the Aβ1-38. These fragments have been identified in CSF, and may be useful biomarkers as well [162]. Thus, measuring the CSF ratio of Aβ42/40 (and other 42/X peptides ratios, e.g., 42/38, etc.) has been attempted. Indeed, the CSF ratio of Aβ42/40 has proven to be a reliable marker of AD, both at the dementia and MCI stage, as well as of the risk of progression from MCI to AD [163,164,165,166]. Furthermore, this ratio was also useful to differentiate AD from other non-AD cognitive changes, such as subcortical deficits of vascular disease. Such correlations were validated by both amyloid PET and extensive clinical and conventional MRI approaches. CSF Aβ42/40 correlates almost completely with amyloid PET, and when there is discordance, longitudinal studies have shown that PET imaging turns positive within a few years [117,119,150]. Finally, it was shown that different assay platforms resulted in similar results [167].

Blood. Serum or plasma measurement of Aβ has become possible and reliable thanks to the increased sensitivity and specificity of the available diagnostic platforms. Initial testing was based on ELISA (enzyme-linked immunosorbent assay), but results have been equivocal at best [148,168]. Interference by plasma proteins was likely significant in these early attempts. ELISA have been progressively improved to reach higher sensitivity, and other platforms have been developed, including SiMoA (Single Molecule Array) [169,170,171], immunomagnetic reduction [172], multiplex assays (e.g., xMAP- [173]), MSD [174] and more. As discussed in more detail below, in order to overcome the complexity of the blood and the interference by blood proteins and heterophilic antibodies and low concentrations of the target biomarker [113], the more modern platforms work with very high dilution of the sample and amplification of the specific signal, allowing for detection of picomolar/mL (or less) concentrations of the target protein [175,176]. Thanks to these advances, we can now reliably measure the Aβ42/40 ratio in plasma.

Studies using SiMoA have, first of all, confirmed a significant correlation between CSF and plasma [170,177] Aβ42, Aβ40 and their ratio, and further confirmed the CSF data on AD and MCI vs. control patients; AD and MCI patients show reduced levels of Aβ42 and Aβ42/40 ratio compared to cognitively intact control subjects, confirmed by amyloid PET.

High-precision assays for plasma Aβ42/40, using immunoprecipitation followed by liquid chromatography-mass spectroscopy (IP–MS) [178], are strongly predictive of brain amyloidosis as validated by benchmark amyloid-β-PET, and reach 90% accuracy [175,179,180]. In one study, a novel fragment ratio to Aβ42 (APP669–711/Aβ42) was also used to predict amyloid-β-PET positivity [175].

Interestingly, in the case of Aβ, another factor that interferes with the accurate plasma measurement is its extra-cerebral production (e.g., platelets, [181]), which is difficult to control. Hence, while it is possible to detect a reduction of the Aβ42/40 ratio in plasma in AD vs. control subjects, plasma reduction only totals about 15%, while in CSF we can see as much as 50% reduction [115,176]. When analyzing the overall trajectory of Aβ variation in plasma vs. CSF, in parallel with Aβ deposition in the brain by PET, by a variety of modern assays, plasma Aβ does not perform as well as CSF [182], showing a narrower range, making it still a less sensitive tool.

### 2.2. Tau

In AD, tau undergoes significant conformational change and abnormal phosphorylation, leading to the formation of inclusions throughout the brain, that start in the entorhinal cortex (EC) and, as the disease evolves, spread progressively throughout the brain, leading to synapse loss and neuronal death, which correlate well with the clinical onset and progression of symptoms [25,183,184]. High levels of tau, both total (t-tau) and phosphorylated (p-tau), have consistently been detected in CSF of AD patients compared to healthy controls [115,185,186,187,188,189,190,191,192]. Studies in FAD and SAD suggest that the tau-PET signal associated with neurofibrillary tangle pathology increases at symptom onset and appears about one decade after CSF soluble tau increase [193,194,195,196]. Other studies suggest that, while neurodegeneration progresses, the rate of increase of p-tau and t-tau levels may actually be reduced [118,133,197], likely in relation to the massive neuronal loss. In this dynamic scenario, where the rate of p-tau accumulation changes, and Aβ may show fluctuations in time, as discussed previously, an ideal biomarker should be specific enough for an adequate pre-clinical detection of AD and to track the disease progression.

As previously reported by Augustinack et al. [183], distinct tau phosphorylation sites correlate with severity of neuronal cytopathology in AD. Pre-tangle state, intra- and extra- neuronal neurofibrillary tangles were used to characterize different stages of pathology. More specifically, p-T231, p-S262, and p-T153 (T: Threonine, S: Serine) were shown to stain non fibrillar tau and were identified as early phosphorylation markers; p-T175/181, p-S262/p-S356, p-S422, p-S46 and p-S214 co-localized with intra-neuronal fibrillar tau structures, and increase as disease progresses; p-S199/p-S202/p-T205, p-T212/p-S214 and p-S396/p-S404 identified instead extracellular tangles composed of substantial filamentous tau structures, seen in later stages of the disease.

In addition to focusing on crucial site-specific phosphorylation, it has been shown, by cryo-EM analysis in AD as well as in other tauopathies, that the most disease-specific change is a tridimensional folding of the tau filaments, which is a signature of the particular disease examined; these tau molecular conformers can in fact differentiate between AD, Pick’s disease and corticobasal degeneration (CBD) [198,199,200,201]. Moreover, an AD-specific epitope formed by two discontinuous portions of tau, _7_EFE_9_ and _313_VDLSKVTSKC_322_ and recognized by the MC1 antibody [198,202,203], represents an early aberrant conformation of tau present both in a soluble form of the protein and in paired helical filaments (PHF) assemblies [204]. Interestingly, the level of MC1 reactivity correlates with the severity and progression of AD. To our knowledge, no assay has been developed to detect this early marker of pathology in biofluids, likely due to the possibility that tau truncated species [205,206,207,208] could prevent the identification of a conformational tau epitope using the currently available immunoassays, which are-based primarily on mid-region-directed antibodies. We believe that, although this pathological feature is not per se easily “translatable”, it should be kept in consideration when designing and developing new tau biomarkers platforms.

In summary, the identification of precise phosphorylated tau species and conformers in the biological fluids will contribute not only to early preclinical diagnosis of AD, but will also allow for a “personalized” approach by staging the disease in its progression and possibly monitoring the effect of therapeutic interventions. Here below is a review of the classic and novel approaches to detect tau and its phosphorylated modifications in CSF and blood.

CSF. Increased phosphorylation and axonal degeneration are believed to induce passive release of tau from the microtubules into the extracellular space, possibly as a neuronal response to Aβ toxicity [209,210]. This process results in augmented levels of t-tau and p-tau in the interstitial fluid and consequently in the CSF. From a neuropathological perspective, analysis of postmortem brains has shown a certain degree of correlation between the neocortical tau burden and CSF p-tau [190,211]. Tau phosphorylated at Threonine 181 (p-T181), t-tau and Aβ42 have been extensively validated in the CSF as biomarkers of AD and are currently widely used as diagnostic benchmarks in clinical and research studies [54,109,212]. CSF p-T181 tau has also proven useful in differential diagnosis of dementia [213,214,215] and to predict cognitive decline in preclinical and prodromal disease stages [213,216,217], with specificity for AD.

An ongoing effort focuses on standardizing protocols and developing criteria for the appropriate use of CSF tau assessment in the diagnosis of AD, with the intent of integrating CSF biomarkers into clinical practice [218,219,220]. In this respect, the distinctive value of the CSF p-T181 tau level has still to be thoroughly demonstrated. In one comparison study, p-T181, tau phosphorylated at Serine 199 (p-S199), and tau phosphorylated at Threonine 231 (p-T231) showed similar diagnostic accuracies for AD [221]. On the other hand, Spiegel et al. [222] reported that CSF p-T231 tau levels are superior to p-T181 in clustering AD subjects from normal control, suggesting the potential for CSF p-T231 assay to be employed in early AD diagnosis. These data are in line with the AD neuropathological picture where p-tau231 shows greater sensitivity for neurofibrillary tangles than p-T181 [211,223], with propensity to accumulate in layer II of the entorhinal cortex [183], known to be the first limbic area in AD consistently targeted by neurofibrillary pathology [224].

Together with p-T181 and p-T231, other phospho-epitopes are under investigation with the hope to find even better AD diagnostics. Barthélemy et al. [225] reported that CSF tau phosphorylated at Threonine 217 (p-T217) is specifically associated with amyloid-β pathology, suggesting a strong interplay between AD amyloidosis and hyperphosphorylation of tau on p-T217; moreover, p-T217 outperforms p-T181 as a biomarker for detecting both the preclinical and advanced forms of Alzheimer’s disease. In a second study, Barthelemy et al. [226] suggested a pattern of tau staging linking CSF site-specific phosphorylation to three different phases of the disease progression. By analyzing FAD, SAD and patients with unaffected cognition but at risk of disease (based on the presence of abnormal Aβ pathology); the authors identified an array of tau phosphorylation uniquely associated with structural, metabolic, neurodegenerative and clinical markers of disease. The study shows that (1) p-T217 and p-T181 rise in parallel to aggregated Aβ as early as 20 years before the development of neurofibrillary tangles, (2) p-T205 correlates with hypometabolism and atrophy closer to symptom onset, and (3) t-tau spikes when cognitive decline manifests. All of these data are in line with another study where the associations of p-T217 with tau PET, CSF and PET measures of amyloid pathology were tested in comparison to p-T181 [220]; the authors found that CSF p-T217 correlates better than p-T181 with PET measures of tau and amyloid pathologies in AD, and more accurately distinguishes AD from non-AD neurodegenerative disorders, suggesting the benefit of employing CSF p-T217 as a biomarker of AD in clinical practice.

Furthermore, investigating tau fragments in CSF, Chen et al. [227] developed a set of tau immunoassays able to detect different populations of tau fragments in CSF and blood. The authors were able to demonstrate that mid-region- and N-terminal-containing fragments increase with disease, while full-length tau is just a small fraction of the tau present in both normal and AD CSF. A recent study has also shown that the ratio between C-terminally truncated tau368 and t-tau is significantly decreased in patients with Alzheimer’s disease, with a strong correlation with ^18^F-GTP1 retention in brain [207] (a measure of tangle pathology in vivo); these data suggest that tau fragment may preferentially accumulate in tangles, and the CSF ratio tau368/t-tau reflects tangle pathology in brain and can be used as an additional tau biomarker to stage and improve the diagnosis of Alzheimer’s disease.

Several studies indicate that TBI can be classified as an independent risk factor for developing Alzheimer’s disease. As previously discussed for Aβ, the axonal injury resulting from the trauma drives the development of both Aβ plaques and NFT [228,229,230,231,232]. Interestingly, not only do tau levels increase in the CSF of the concussed patients undergoing repetitive trauma (i.e., sports players), but serum t-tau levels have been reported to increase in the acute stage following the concussion, while decreasing during rehabilitation [230,233,234]. Similarly, serum p-tau (p-T231 and p-S202) levels, though reduced, were shown to be still higher compared to the control levels even at six months post-injury [235].

When considering tauopathies different from AD with significant NFT pathology and neurodegeneration, CSF p-T181 and t-tau levels do not necessarily increase; this is the case for progressive supranuclear palsy (PSP) and some forms of FTD [221,236,237,238]. Why increased CSF t-tau and p-tau are specific to AD can be explained by considering that (1) the pattern of tau phosphorylation occurring in these disorders is disease-specific, (2) tangle pathology and neurodegeneration in AD may be consequent to Aβ accumulation, with disease-specific molecular cascades, such as kinases and proteases activation, (3) disease-specific tau truncation could prevent detection by currently available assays, and (4) specific pathological changes can be more severe in AD than in other tauopathies.

Blood. Until recently, AD biomarkers’ quantification was limited to the CSF. However, while combining CSF biomarker analyses and PET scans have the potential to improve diagnostic accuracy, these methodologies show limitations, impeding their use as first-line diagnostics [113]. Developing an accurate and standardized blood test will be more cost effective than PET imaging [239,240], less invasive than CSF testing [241] and faster. Hence, blood-based biomarkers represent next-generation diagnostics for AD and other CNS diseases. One matter of concern is that blood tau has the potential to derive from other sources in addition to neuronal tau [242,243]. In this respect, novel ultrasensitive detection technologies have been developed to facilitate the detection of very low concentrations and variations of tau in the blood [186,244,245] using very high dilutions of the biological fluid: employing these technologies has pushed forward the potential for plasma tau to serve as a biomarker for neurodegeneration in AD, avoiding the interference of non-neuronal tau.

Longitudinal studies originally showed that higher levels of plasma t-tau are associated with greater cognitive decline and risk of MCI [246,247,248], with no correlation with elevated brain Aβ [247]. As discussed, plasma t-tau assays can identify neuronal injury in acute brain disorders, such as TBI, but work relatively poorly for AD diagnosis, and the correlation with CSF t-tau is weak [249]. In this context, t-tau could be intended as a non-specific prognostic marker for dementia and related endophenotypes [248]. Despite the lack of correlation between plasma and CSF t-tau, Fossati et al. [186] have recently demonstrated that by combining plasma t-tau with CSF t-tau or CSF p-T181 tau, the accuracy for the differentiation of AD versus cognitively normal controls increases, suggesting that plasma tau may be a useful biomarker to increase diagnostic power and determine therapeutic efficacy in clinical studies.

Analysis of phosphorylated tau in blood has so far been focused on the development and validation of assays for p-T181 [182,246,250,251,252,253], demonstrating that increased plasma p-T181 (1) correlates with amyloid and tau PET measures [250,254,255], allowing the identification of individuals with Aβ pathology but tau PET-negative measurements [253], (2) goes in parallel with CSF p-T181 [182,255], and (3) can help in the differential diagnosis of non-AD types of dementias [253,254,255].

While writing this review, two groups published strong data in support of the use of a blood-based assay detecting p-T217 tau. First, Barthelemy et al. [256] developed a protocol of purification and enrichment of tau from plasma, followed by liquid chromatography (LC-MS); this system (1) allows reliable detection of p-T217 showing high correlation with the CSF measurements, and (2) confirms the ability of this biomarker in clustering amyloid positive and negative patients. The limitation of this strategy is the high volume of blood needed to purify and extract tau and the elaborate methodology, not compatible with the idea of developing a fast and easy-to- use assay. In the second study, Palmqvist et al. [256] employed the Meso Scale Discovery (MSD)/Lilly platform (described below) and validated the value of p-T217 in plasma to discriminate Aβ+ MCI and AD patients from other neurodegenerative disorders and controls, including sensitive discrimination of autosomal-dominant pre-symptomatic Aβ+ patients. In all, given the high potential of measuring CSF p-T217 previously described, it will be of great interest to follow the development and validation of novel ultrasensitive platforms aimed at detecting p-T217 in blood, employing fully automated platforms to integrate this biomarker in clinical practice. Similarly, setting up sensitive and specific assays able to detect p-T231 (early phosphorylated epitope) and p-S396/404 (late phosphorylated epitope) in blood will also be relevant to stage the AD pathology and to monitor future therapeutic interventions.

Finally, an ultra-sensitive assay was developed in blood, to detect an N-terminal tau fragment (NT1) [227], showing that N-terminal fragments of tau mapping in the 6–198 sequence are increased in AD and MCI patients and may be useful as a screening blood-based test for AD. This approach is extremely significant due to the possible generation of disease-specific tau fragments (as for APP and Aβ), and will need further investigation.

Immunoassays and ultrasensitive platforms to detect tau in biological fluids. Several assays have been tested and extensively validated in CSF and plasma. In Table 1 we select and catalogue the most used assays in the tau biomarker field at the moment, with emphasis on their specificity, fluid samples’ applicability and limit of detection. The list of assays includes (1) standard and second generation ELISAs (INNOTEST, EUROIMMUN, Elecsys), (2) MSD platforms (3) Single-Molecule enzyme-linked immunosorbent assay (SiMoA), (4) Mass spectrometry analysis, (5) superconducting-quantum-interference-device Immunomagnetic reduction (SQUID-IMR). The detection range spans from few pg/mL to fg/mL according to the sensitivity of the assay. EUROIMMUN and Elecsys are automated second-generation immune assays that have demonstrated good analytical performance with clinically relevant measuring ranges, supporting their use in clinical trials and practice [257,258]. MSD technology with electrochemiluminescence detection comes with several advantages compared to standard immunoassays, including high sensitivity, low background and wide dynamic range of detection [174]. Notably, Palmqvist et al. [182] recently ran a comparative analysis of Aβ and tau CSF assays from five different manufacturers: Elecsys (Aβ42, Aβ42/40, t-tau, p-tau181) EUROIMMUN (Aβ42, Aβ42/40, t-tau, p-tau181), INNOTEST (Aβ42, Aβ42/40, t-tau, p-tau181), MSD (Aβ42, Aβ42/40, t-tau) and Lilly (p-tau181, p-tau217): Aβ42, Aβ42/40, p-tau and t-tau had very similar trajectories between assays.

Moving to ultra-sensitive platforms, Rissn et al. previously developed [169,259] a Single-Molecule enzyme-linked immunosorbent assay (SiMoA) that detects serum proteins at sub-femtomolar concentrations, restricting the fluorophores produced by individual enzymes to considerably small volumes, hence capturing the fluorescence of each single molecule. This methodology has been extensively used to improve t-tau and p-tau detection in CSF and plasma (Quanterix).

SiMoA and INNOTEST ELISA have been also demonstrated to strongly correlate in CSF t-tau in a comparison study [186]. Moreover, in a recent study, Karikari et al. [253] validated a blood-based immunoassay measuring p-T181 using a sandwich immunoassay format on SiMoA technology.

To further quantify tau at high sensitivity, Barthelemy et al. has developed a protocol to detect tau and p-tau in CSF and plasma employing targeted high-resolution mass spectrometry (MS) [225,226,256,260,261]. Additionally, superconducting-quantum-interference-device Immunomagnetic reduction (SQUID-IMR) has proven efficient at quantifying t-tau and p-T181 in plasma in early stages of AD [172,252].

## 3. Biomarkers of Synaptic and Neuronal Loss

Synaptic loss first and overt neuronal demise later are fundamental aspect of AD pathology and are, together with tau deposition, the real correlate of the manifestation of symptoms. Neurodegeneration is the “N” in the ATN system, and is a fundamental correlate of the functional disability of AD patients. When patients present to their doctors with memory loss or other cognitive changes, due to AD, it is estimated that a large number of neurons have already been lost to neurodegeneration, but recent models are looking at the effect of more subtle synaptic loss as an inciting event responsible for symptoms [70]. In this context, several markers of synaptic disruption have been proposed as indicators of disease onset and progression.

### 3.1. Neurofilament Light Chain (NfL)

NfL belongs to the type IV intermediate filaments, which make up the neuronal axoskeleton, maintain neuronal caliber and play a role in intracellular transport to axons and dendrites. With the neurodegenerative process, in AD as in other disease [262], NfL is released in the extracellular space. NfL has been detected in both CSF and blood, and appears to be extremely sensitive to AD onset and progression, predicting Aβ PET positivity as well; furthermore, longitudinal studies in familial AD, have shown accurate prediction of disease onset, as far as 10 years prior to symptoms [263,264,265,266]. The tight correlation between CSF and blood values [267] make NfL one of the most reliable blood biomarkers to predict onset and course of neurodegeneration.

### 3.2. Neurogranin

Neurogranin (Ng) is a neural-specific postsynaptic protein, concentrated on dendritic spines, in the hippocampus and basal forebrain. Given its localization at the epicenter of the pathogenic events in AD, it is not surprising that it would be released in the extracellular space and be detected in CSF. In AD, CSF levels of Ng grow quite rapidly prior to the presentation of cognitive deficits, before NfL does, in an Aβ-dependent way [268]. Plasma levels do not seem to be as reliable as CSF, possibly due to peripheral production [269]. Importantly, and contrarily to NfL, other neurodegenerative disease do not show an increase of Ng, raising the possibility of an AD-specific synaptic effect [185].

Other biomarkers in this category are being investigated, such as NSE and SNAP-25, and are the subject of extensive analysis, but are mostly used in complex and integrated predictive models [133,270] and will not be reviewed here.

## 4. Biomarkers of Inflammation and Microglia “Dysfunction”

Inflammation, microglia and astrocytes activation are well-recognized features of AD pathology, as for other neurodegenerative diseases. It is unclear if they are integral part of the original pathogenesis or are reactive features to the deposition of amyloid and tau. To really understand this aspect, we should investigate, with an unbiased approach, the very early features of patients with a predisposition to develop AD pathology, for example in large autosomal dominant cohorts, in prospective longitudinal studies, and some effort is under way in this context. For the time being, we will focus on the data available from the earliest detectable stages of AD pathology, the prodromal AD cases.

### 4.1. Soluble TREM2

The Triggering Receptor Expressed on Myeloid Cells 2 (TREM2) is a transmembrane receptor, belonging to the family of cell surface transmembrane glycoproteins [271] which mediates signaling and cell activation following ligand binding. It is expressed and functions in subsets of myeloid cells, e.g., granulocytes, dendritic cells, macrophages, and microglia in the brain [272,273,274,275]. Among its complex and protean roles, its function is to regulate immune cells’ activation upon binding of a variety of ligands [66], including APOE and Aβ. In the brain, where it may be expressed preferentially in some regions, such as the hippocampus, and differentially in response to the pro/anti-inflammatory environment, it mediates microglia activation, proliferation, migration, apoptosis and expression of pro-inflammatory cytokines, in particular after binding with aggregated proteins such as Aβ, and facilitates the phagocytosis of apoptotic neurons [276]. TREM2 expression is increased in many neurodegenerative conditions, stroke, trauma and even ageing. Rare variants of TREM2, discovered by GWAS studies [277,278], have been associated with the risk of developing AD, in the range of the risk conferred by APOE4, of 2- to 4-fold (heterozygous). The most common AD-associated variant recognized in populations of Europeans descent, but not in Chinese and African-Americans, is the R47H [277,279]. This TREM2 mutant is associated with earlier onset of disease and higher CSF tau levels [280] and seems to demonstrate reduced activity in ligands-binding and hence signal transduction, with a net result of reduced microglia activation and, in AD models, less clearance of Aβ [274]. Given this premise, it is only natural to look at TREM2 as a possible biomarker for AD. The soluble form of TREM2 (sTREM2) is found in both CSF and blood [281,282]. In CSF, sTREM2 is elevated in AD patients vs. controls, with positive association with t-tau levels [212,283]. In familial AD, sTREM2 CSF levels increase prior to clinical onset, but after detectable changes of Aβ and tau in CSF [284]; this is a moment in time where the brain is already at an advanced stage of pathology, in particular tau, with significant neuronal loss and when patients are soon going to lose their cognitive reserve and show symptoms. From a clinical perspective, this is relatively late to be useful as early prediction of disease or point of intervention, but from a pathogenic point of view, it may signify a “breaking point” of the Aβ and tau pathology containment system, where microglia cannot keep up with plaques and tangles any more. This is extremely important to consider in the development of therapeutic strategies in the future. More work is needed to create more sensitive assays in order to detect sTREM2 blood changes, as early as possible. Additionally, since the degree of inflammation and microglia activation differs from patient to patient, and secondary to treatment (e.g., anti-amyloid strategies), a sensitive blood sTREM2 assay may be a very useful tool to monitor phenotypic variants and adverse events form upcoming treatments.

### 4.2. YKL-40

YKL-40 is a glycoprotein expressed in both astrocytes and microglia, and is the protein product of the CHI3L1 gene, a.k.a., Chitinase 3 Like 1. YKL-40 actually lacks chitinase activity and is secreted by activated macrophages, chondrocytes, neutrophils and synovial cells, and plays a role in inflammation and tissue remodeling [285,286,287,288]. The first indication that YKL-40 may be relevant for AD came from an aprioristic evaluation of potential novel AD biomarkers [289]. Since then, several studies have now looked at the both CSF and plasma levels of this protein. YKL-40 is elevated in AD vs. control subjects [106,289,290], although it was also significantly elevated in patients with FTD [291]. Similarly, in MCI, baseline higher levels of YKL-40 were associated with significant more risk to progress to AD [106,289] with a tendency to continue to raise with disease progression [182]. The CSF ratio of YKL-40/Aβ42 was shown to be predictive of the onset of cognitive symptoms [289]. Plasma levels of YKL-40 have shown similar trends, but further validation may be needed to confirm reliable use [292]. In all, YKL-40, with its trend toward increase with disease progression, can be an associated marker for progression and prognosis.

### 4.3. Other Biomarkers of Inflammation and Glial Activation

Several pro- and anti-inflammatory factors have been looked at in both CSF and blood, including IL1, IL6, TNFα, VILIP-1, sTNFR and IP-10 [293,294,295], with some correlation to AD and MCI. Their significant is still unclear, and some are now part of multiplex panes, used in association with specific AD markers, arguably as prognostic indicators of disease progression.

GFAP (glial fibrillary acidic protein) is one of the major intermediate filament proteins of mature astrocytes, and it is released by astrocytes during neurodegeneration [296]. It is not properly a marker of inflammation, but it is a sign of a reactive brain milieu, as astrocytes tend to proliferate in response to tissue injury, and eventually release large amounts of GFAP in the interstitial fluid. Recently, the astrocytic response to brain damage and the inflammatory microglia has been characterized [297,298,299], and is very relevant in the context of AD pathology. The currently available CSF GFAP data show a correlation between increased levels and Aβ and tau evidence of AD [115,300], and recent data on plasma GFAP showed increased concentrations in late and early onset AD patients compared with cognitively normal controls [301]. Given the non-specific nature of GFAP, its use may be in the future confined to monitoring disease onset and progression, and possible response to treatment.

In all, given both the convergent and divergent nature of the inflammatory response, which involves a myriad of molecules and pathways, leading to a fine balance between protective and deleterious inflammation, and the fact that inflammation is an unavoidable element of ageing, any single marker would not be useful in isolation. Rather, a comprehensive approach using combinations of such factors, associated with specific marker of AD and markers of synaptic/neuronal loss (see below), has the best chance to be useful in clinical practice and research.

## 5. Biomarkers of Other Associated Pathology

### 5.1. TAR DNA-Binding Protein 43 (TDP-43)

TDP-43 can be present as co-pathology in AD (up to 50% of cases), and is the main feature of other neurodegenerative diseases, such as FTD and ALS. Its role is that of a transcriptional repressor, which regulates genes’ alternate splicing. An extensive study [302] was able to link the presence of TDP-43 in AD brains with the likelihood of clinical AD presentation; but to date, a reliable CSF or blood marker is not available, although notable attempts and data are present for ALS [303,304]. Part of the issue is the non-specificity of TDP-43 accumulation, as the concept of a spectrum of TDP-43 neurodegenerative presentation is becoming more evident [305]. In this context, TDP-43 may be useful as a biomarker in the future to predict course of disease or variants of AD presentation.

### 5.2. Alpha-Synuclein

Alpha-synuclein is the key component of Lewy bodies, and is classically found in Parkinson’s pathology, LBD, multi-system atrophy (MSA) and AD (also 50% of cases) variants. It is involved in presynaptic signaling, inhibiting phospholipases and moderating vesicles transport at the terminals [306]. As for TDP-43, despite the feasible measurement of α-synuclein in CSF and blood, no significant correlation has been made to date, likely due to the non-specific nature. What is different here is the possibility to harness α-synuclein’s specific tendency to aggregate in a “prion-like” fashion [307,308], for example by CSF RT-QuIC (real-time quaking-induced conversion), likely in virtue of its conformational changes [309]. Further studies will have to determine the sensitivity of the test for AD, as being able to detect synucleinopathy in AD cases would be highly significant for prognostic, therapeutic and research purposes.

## 6. Vascular Damage and the Blood Brain Barrier

Vascular damage is an increasingly recognized pathological component of AD, both as an independent consequence of the toxicity of Aβ and tau [310,311], and as a frequent co-morbidity [312,313] with microvascular disease. The diagnosis if “mixed dementia” is ever more frequent, as we scan more patients in our clinical practice. Although the real significance of the interplay between amyloid/tau pathology and the microvascular damage is still unclear, as the basic pathological mechanisms are quite complex, we have been able to detect vascular damage and blood-brain barrier (BBB) leak [314,315] in vivo. Modern magnetic resonance imaging (MRI) techniques, such as dynamic contrast-enhanced (DCE)-MRI, can show BBB permeability in specific regions, i.e., the hippocampus [315,316]. This abnormal permeability seems to be proportional to ageing [317], and is more frequent in MCI and AD patients compared to unimpaired control subjects [315,318,319]. An interesting correlate of MRI studies is the use of a marker of brain capillary damage, the soluble platelet-derived growth factor receptor-β (sPDGFRβ), which is highly expressed in brain capillary pericytes and vascular smooth muscle cells [315], and is shed in the CSF after brain injury, possibly in relation of the activation of proteases such as ADAM10 [320]. Emerging data show that ageing and cognitive decline, independently of AD pathology, are associated with increased CSF levels of sPDGFRβ, which correlate with MRI permeability [317]. Although the use of BBB permeability diagnostics is still in its infancy, and the specificity for AD pathology is unclear, it would certainly be a precious prognostic tool in the hands of clinicians. In this context, and somehow beyond the scope of the current manuscript, the use of gadolinium-free MRI techniques to assess BBB permeability (K-trans) would be extremely welcome.

## 7. Multi-Target Platforms

With so many biomarkers coming onto the scene, and in order to advance both the diagnostic and prognostic assessments, a new generation of assays is under development with the goal to simultaneously catch multiple targets within the same fluid sample. Using such types of assays will be extremely informative in order to identify AD co-pathologies, characterize disease progression and monitor treatment response. Table 2 contains a list of the more popular, commercially available, multi-target platforms.

The NeuroToolKit, in development with Roche’s Elecsys electroluminescence immunoassay platform [300], includes (1) α-synuclein as a marker of synaptic dysfunction, (2) S100b, YKL-40, and glial fibrillary acidic protein (GFAP) as markers of astrocyte activation, (3) sTREM2 and IL-6 as markers of microglial activation and inflammation, and (4) NfL and neurogranin as markers of axonal injury and synaptic dysfunction.

Simultaneous measurement of Aβ42, t-tau and p-T181 in CSF has been previously developed by the xMAP technology [321]. The MILLIPLEX^®^ map multiplex immunoassay kit is a 4Xplex panel enables the simultaneous measurement of Aβ1-40, Aβ1-42, t-tau and p-T181 in human CSF.

The NEUROLOGY 3-PLEX A (SiMoA, Quanterix) detects t-tau, Aβ1-40 and Aβ1-42 in both CSF and blood.

The NEUROLOGY 4-PLEX A (SiMoA, Quanterix) detects t-tau, NfL, GFAP and ubiquitin carboxyl-terminal hydrolase L1 (UCH-L1) both in CSF and blood.

A new 4-PLEX assay has been recently developed (SiMoA, Quanterix) with the ability to target Aβ1-40, Aβ1-4, GFAP and NfL in both CSF and blood (Alzheimer Association International Conference, 2020. Available online: https://alz.confex.com/alz/20amsterdam/meetingapp.cgi/Paper/43506. Accessed on 29 July 2020), and was validated against the parent assays. We await studies on larger number of patients, with diagnostic and prognostic implications.

Using a single platform with the ability to test multiple targets, such as in this case, would be invaluable in clinical practice, given how precious CSF and even blood samples are. Ideal kits should include markers specific of disease (e.g., Aβ42/40 ratio, p-T217 tau) and biomarkers of severity of disease, as NfL, sTREM2 etc.

## 8. Immunoassays, Ultrasensitive Platforms and Their Potential Limitations

One main concern in the development of an immunological assay is to exclude any factor that could potentially (1) interfere with the detection of the analyte and (2) alter the antibody binding. The first category includes pre-analytical factors (sample storage, anti-coagulants), anti-analyte antibodies, and hormone binding proteins that can change the measurable concentration of the analyte in the sample; the second group consists instead of antibodies such as heterophile antibodies, human anti-animal antibodies (HAAA), and human anti-mouse antibodies (HAMA) which are found in the biological fluids and are able to interfere with the antibody binding in the assay [322]. Unlike plasma (~7 g protein/100 mL) the CSF has only ~0.025 g protein/100 mL—mainly albumin [323]. Given the different composition of CSF and blood, it is relatively easy to anticipate the different susceptibility to assay interference of these two different biological fluids. While the CSF is a relatively “clean” fluid with respect to protein concentration, plasma contains heterophilic antibodies and other molecules that might interfere with the measurements of a biomarker of interest [245]. The presence of HAMA in human plasma has been described in 30% of the population, and consequent interference by human anti-globulin antibodies in immunoassay has been reported in several studies [324,325,326,327,328]. For these reasons, detecting biomarkers for CNS disease in blood not only calls for highly sensitive and specific assays, but require to efficiently remove all the potential confounders. Different methods for the reduction of heterophile antibody and anti-animal interferences in immunoassays have been previously described and include ways to remove or block the interfering antibody [322,329,330]. In this respect, our group has previously developed a protocol combining NaOAc (sodium acetate) and heat-treatment to ‘purify’ tau from the blood of transgenic animals undergone tau immunotherapy [328]. This protocol was validated using mouse and human serum. While the NaOAc/heat-treatment did not affect tau reactivity on CSF samples, human plasma samples displayed an impressive reduction of the apparent tau signals after undergoing the heat extraction protocol. The use of the ultrasensitive assays described here has overcome some of these technical issues. In addition, the use of blockers against heterophilic antibodies in the sample diluent is also helpful to achieve reliable quantification [245]. We still believe that the previous considerations need to be considered, especially when analyzing human samples derived from patients undergone immunotherapy, where the risk of assay interference could compromise the interpretations of the disease course and therapy.

Ageing is per se associated with a reduction of the integrity of the BBB, due to a multitude of causes, e.g., prior infections, general inflammatory state, trauma, tumors and more [315,316]. MCI, AD and cognitive changes in ageing have been associated with non-specific BBB permeability in certain brain regions [317]. The resulting permeability may result in falsification of CSF data for reasons analogous to the blood.

## 9. Novel Approaches to Unbiased Biomarker Discovery: The Case for Metabolomics

A successful preclinical diagnosis of AD may benefit from an unbiased metabolomic analysis of biological fluids. Given that CSF metabolites reflect brain changes better than blood, CSF biomarkers are widely used in research and clinical practice [331]. The strength of metabolomics is in its ability to measure a plethora of metabolites in an unbiased manner, providing a snapshot of an individual’s biological status [331,332]. The ideal system would include a CSF metabolomic profile covering the whole lifespan of individuals belonging to both FAD and SAD cohorts, followed by plasma validation. While this analysis would require decades in human AD, or the longitudinal analysis of autosomal dominant families, running such studies on faithful animal models would make for an optimal surrogate, taking advantage of novel knock-in models, as opposed to transgenic ones, which reproduce the exact genetic defect found in humans [333,334,335,336,337,338].

Metabolomic studies have been performed in CSF, blood and post-mortem tissue from AD and MCI patients [331,339,340]. For example, elevated CSF glutamate and glutamine levels have been described in AD before [332,341,342], and other studies have shown blood and CSF metabolites changes in AD [343,344,345], some correlating with progression and risk of MCI to progress to AD [346]. Most identified pathways relate to energy metabolism, Krebs cycle, mitochondrial function, neurotransmitter and amino acids metabolism, and lipid biosynthesis. Longitudinal studies in FAD cases would be welcome in the near future to understand both the pathological relevance of the said pathways and their power to predict AD.

## 10. Discussion and Conclusions

Several studies have by now assessed the use of isolated biomarkers vs. their combinations to predict diagnosis of AD, or progression of MCI to AD. No single biomarker, in CSF, blood, imaging or cognitive alone is capable to predict onset and course reliably. Rather, the combination of multimodal biomarkers is required to reach reliable predictions [100,300,342,347,348]. CSF Aβ1-42 reduction (best proxied by the Aβ42/40 ratio) starts very early before symptoms onset, peaks a few years later when amyloid PET is at the zenith; CSF tau elevation follows Aβ, and represent clinical disease severity very well. NfL increase starts early, before tau, and is a very good indicator of neuronal damage early in the disease, though it is not specific for AD; still, its reliability in blood as well in CSF, makes it an excellent marker to track disease progression and possible response to therapies. Similarly, sTREM2 and YKL-40 are excellent markers of inflammation, and although their best use is in CSF, they could be extremely useful to monitor possible adverse events of therapeutic intervention. Aprioristic analysis of CSF and blood by metabolomic, proteomic, lipidomic and other large-scale approaches could be the key, if done early enough in the pathogenic course, to generate hypothesis on the primordial triggers of the disease, besides to identify AD patients and predict their trajectory. In this respect, autosomal dominant families and patients with Down’s syndrome [134,349,350] may be the ideal subjects for dedicated longitudinal studies.

Given this premise, the biomarker field has to drive every resources toward the development of blood-based assays able to provide high specificity and sensitivity to excel in both diagnosis (Aβ, and tau) and prognosis (i.e., NfL) of AD. Implementing AD biomarkers in clinical practice will require blood-based assays (1) easy-to-use in terms of human specimen collection (few mL of blood) and methodology, (2) with high sensitivity (under the pg/mL threshold) and specificity, (3) free of interference produced by several confounders associated with the ageing process. The focus on pre-symptomatic Aβ+ populations is paramount (e.g., autosomal-dominant cohorts) if we really want to diagnose the disease in a phase where significant intervention is still possible.

## Figures and Tables

**Table 1:** Immunoassays and ultrasensitive platforms to detect tau in biological fluids.

Type of Assay	Platform/ Manufacturer	t-tau	p-tau	Fluid Sample	LOD pg/mL	References (PMID/link)
**Standard ELISA**	INNOTEST Fujirebio	Total tau Antibodies: Capture HT7/AT120 Detector BT2		CSF	34	8748926
	INNOTEST Fujirebio		p-T181 Antibodies: Capture HT7 Detector AT270	CSF	13	10788705
	Applied Neurosolutions (Dr. Peter Davies)		p-T231 Antibodies: Capture CP27 Detector CP9	CSF	9	26444757
**Automated ELISA**	Elecsys	Total tau Antibodies: Not available		CSF	63	31129184
	Elecsys		p-T181 Antibodies: N.A.	CSF	4	31129184
	EUROIMMUN	Total tau Antibodies: Capture ADx201 Detector ADx215		CSF	44.2	27447425
	EUROIMMUN		p-T181 Antibodies: N.A.	CSF	N.A.	27447425
**MSD-ECL**	MSD		p-T181 Antibodies: Capture biotinylated AT270 Detector SULFO-TAG-LRL (anti-tau, Lilly)	Plasma	N.A.	29626426
	MSD/Lilly		p-T181 Antibodies: Capture biotinylated AT270 Detector SULFO-TAG-LRL (anti-tau)	CSF	N.A.	31709776
	MSD/Lilly		p-T217 Antibodies: Capture biotinylated IBA413 Detector SULFO-TAG-LRL (anti-tau)	CSF	N.A.	31709776
	MSD/Lilly		p-T217 Antibodies: Capture Biotinylated-IBA493 Detector SULFO-TAG-4G10-E2 (anti-tau)	Plasma	N.A.	31709776
**SiMoA**	Simoa™ Tau 2.0 kit HD-1 platform (Quanterix)	Total tau Antibodies: Capture sequence AA16–AA24 Detector sequence AA218–AA222		CSF	0.019	101444
	Janssen R&D Simoa technology HD-1 platform (Quanterix)	Total tau Antibodies: Capture HT7 Detector PT82		CSF	N.A.	32246036
	Modiefied version of Simoa™ Tau 2.0 kit HD-1 platform (Quanterix)		p-T181 Antibodies: Capture sequence AA16–AA24 Detector AT270	Plasma	0.0090	28866979
	Sandwich immunoassay format on Simoa technology (Quanterix)		p-T181 Antibodies: Capture AT270 Detector Tau12	Plasma Serum	N.A.	32333900
	Simoa™ p-181 kit HD-1 platform (Quanterix) NEW		p-T181 Antibodies: N/A	CSDF Plasma Serum		Alzheimer Association International Conference, 2020. Available online: https://alz.confex.com/alz/20amsterdam/meetingapp.cgi/Paper/41238. Accessed on 29 July 2020.
	Janssen R&D Simoa technology (Quanterix)		p-T217 Antibodies: Capture PT3 Detector PT82	CSF	N.A.	32246036
	Simoa^®^ pTau-231 Advantage Kit HD-1 platform (Quanterix)		p-T231 Antibodies: Capture AT270 Detector Tau12	CSF	0.621	Quanterix.com. Available online: https://www.quanterix.com/sites/default/files/assays/Simoa_pTau-231_Data_Sheet_HD-1_HD-X_Rev02.pdf. Accessed on 29 July 2020.
	NT1 assay Simoa technology HD-1 platform (Quanterix)	N-terminal tau Antibodies: Capture Tau12 Detector BT2		CSF Plasma	0.2-0.7	30419228
**Nano liquid chromatography-HRMS**		Total tau	p-T181 p-S202 p-T205 p-T217	CSF	N.A.	26742856 32161412
**(IP)-LC-MS**			p-T217 p-T181	Plasma	p-T217:0.05 p-T181:0.2	32725127
**SQUID-IMR**		Total tau	p-T181	Plasma	N.A.	29376870

A detailed list of the original and the more updated assays used in the tau biomarker field is provided, with emphasis on total, phosphorylated or truncated tau. Type of assays and platforms: ELISA (Enzyme-linked immunosorbent assay); Meso Scale Discovery (MSD) technology with electrochemiluminescence; SiMoA: Single-Molecule enzyme-linked immunosorbent assay; Nano liquid chromatography–HRMMSD-ECLS: Nano liquid chromatography–high resolution mass spectrometry; (IP)-LC-MS: immunoprecipitation coupled with liquid chromatography-spectrometry; SQUID-IMR: superconducting-quantum-interference-device Immunomagnetic reduction. Fluid samples are specified (CSF, plasma, serum). Limit of detection (LOD) of the assay is provided where available, if not it is marked as N.A. References are listed as PMID or hyperlink.

**Table 2:** Multi-target platforms.

Type of Assay	Platform/ Manufacturer	Targets	Fluid Sample	LOD pg/mL	References (PMID/link)
**SiMoA**	Simoa^®^ Neurology 3-Plex A Kit HD-1 platform (Quanterix)	t-tau Aβ40 Aβ42	CSF plasma	Tau: 0.019 Aβ40: 0.196 Aβ42: 0.045	Quanterix.com. Available online: https://www.quanterix.com/sites/default/files/assays/Simoa_N3PA_Data_Sheet_HD-1_HD-X_Rev04%20%281%29.pdf. Accessed on 29 July 2020
	Simoa^®^ Neurology 4-Plex A Kit HD-1 platform (Quanterix)	t-tau NfL GFAP UCLH-1	CSF plasma	Tau: 0.024 NfL: 0.104 GFAP: 0.221 UCLH-1: 1.74	Quanterix.com. Available online: https://www.quanterix.com/sites/default/files/assays/Simoa_N4PA_Data_Sheet_HD-1_HD-X_DS-0074_rev7.pdf. Accessed on 29 July 2020
	Simoa^®^ 4X-plex neurology Kit (Quanterix) NEW	Aβ40 Aβ42 GFAP NfL	CSF plasma	Aβ40: 0.084 Aβ42: 0.148 GFAP: 1.66 NfL: 1.157	Alzheimer Association International Conference, 2020. Available online: https://alz.confex.com/alz/20amsterdam/meetingapp.cgi/Paper/43506. Accessed on 29 July 2020.
**Luminex^®^xMAP^®^**	The MILLIPLEX^®^ map 4X multiplex immunoassay kit (Millipore, Sigma)	t-tau p-T181 Aβ40 Aβ42	CSF	N.A.	Emdmillipore.com. Available online: https://www.emdmillipore.com/US/en/product/MILLIPLEX-MAP-Human-Amyloid-Beta-and-Tau-Magnetic-Bead-Panel-Multiplex-Assay,MM_NF-HNABTMAG-68K. Accessed on 29 July 2020.
**NeuroToolKit**	Roche’s Elecsys electroluminescence immunoassay platform	t-tau, p-T181 Aβ40, Aβ42 α-synuclein S100b YKL-40 GFAP sTREM2 IL-6 NfL Neurogranin	CSF	N.A.	32573951

Listed are some of the assays available for the simultaneous detection of multiple biomarkers in CSF or plasma. Type of assays and platforms: SiMoA: Single-Molecule enzyme-linked immunosorbent assay; Luminex XMAP: bead-based multiplex immunoassay in a microplate format; NeuroToolKit: based on electroluminescence immunoassay platform. Targets and Fluid samples are specified. Limit of detection (LOD) of the assay is provided when available. References are listed as PMID or hyperlink.
